# Optimizing multi‐trait selection for profitability in Illinois wheat using a factor analytic mixed model and an economic selection index

**DOI:** 10.1002/tpg2.70313

**Published:** 2026-09-30

**Authors:** Lucas Berger Munaro, Daniel J. Tolhurst, Jessica E. Rutkoski

**Affiliations:** ^1^ Department of Crop Sciences University of Illinois Urbana‐Champaign Urbana Illinois USA; ^2^ The Roslin Institute and Royal (Dick) School of Veterinary Studies The University of Edinburgh Midlothian UK

## Abstract

Economic selection indices can improve the efficiency of selection for multiple traits, but they are underutilized in plant breeding due to limited research on the economic values of traits and a lack of well‐documented case examples employing economic indices. This study evaluated a genomic‐based Smith–Hazel index incorporating empirically derived economic weights for grain yield, test weight, and maturity in soft red winter wheat (*Triticum aestivum* L.) in Illinois. Economic weights used in the index were estimated using a function describing expected profit considering the predominant cropping system and grain quality pricing scheme of the region. Data from 2995 genotypes, five traits, and 12 environments (2022–2024) were analyzed together with genome‐wide marker data using a factor analytic (factor analytic model with *k* factors [Fa*k*]) model fitted to the environment‐by‐trait combinations to obtain predictions of breeding value. The optimal multi‐trait multi‐environment (MTME) model (FA5) explained over 91% of the additive genetic variance and achieved high prediction accuracy (≥0.95). Compared with a single‐trait multi‐environment (STME) model, use of an MTME model for index selection increased predicted economic return by 3.4% ($120 ha^−1^ vs. $116 ha^−1^ for STME). Furthermore, index‐based selection under MTME yielded a predicted net merit gain of $120 ha^−1^, compared with $97.5 ha^−1^ when selecting on grain yield alone, demonstrating the economic value of multi‐trait selection. Covariance estimates from the MTME model indicated that the genetic correlations between traits depended upon the environment, likely due to environment‐specific stressors. Overall, we determined that an optimal economic selection index can be constructed for a regionally focused plant breeding program using an FA*k* model and economic weights.

## Plain Language Summary

The use of economic selection indices can improve the effectiveness of multi‐trait selection for the purpose of improving profitability; however, these indices are underutilized in plant breeding programs. This study determined the economic weights for three key traits in soft red winter wheat in Illinois and used these weights with predicted breeding values to compute an economic selection index. Predicted breeding values suitable for the index were obtained using University of Illinois wheat breeding program data. Two statistical models were compared for their impact on gain from index selection. Response to index and single‐trait selection was also compared. Overall, we found that selection based on the index and modeling traits as genetically correlated improved expected response in profitability.

**Abbreviations**
Adv: Addieville
DON: deoxynivalenol
FA: factor analytic
FHB: Fusarium head blight
GBLUP: genomic best linear unbiased prediction
GEBV: genomic estimated breeding value
GEI: genotype‐by‐environment interaction
GET: genotype‐by‐environment‐by‐trait
GY: grain yield
HD: heading time
MET: multi‐environment trial
MTME: multi‐trait multi‐environment
Neo: Neoga
REML: residual maximum likelihood
SHI: Smith–Hazel index
SRWW: soft red winter wheat
STME: single‐trait multi‐environment
Stp: St. Peter
TPE: target population of environments
TW: test weight
Urb: Urbana

## 1 INTRODUCTION

The primary goal of an applied plant breeding program is to develop and release improved commercial varieties or hybrids. This objective is typically achieved through two processes: population improvement and product development (Gaynor et al., 2017). Population improvement focuses on increasing the population mean breeding value for traits of importance, while product development focuses on identifying and advancing superior breeding materials for cultivar release. Population improvement is achieved by conducting multiple cycles of breeding in which the breeding materials are evaluated, and individuals identified to be the best parents are intermated to generate new breeding materials that themselves become candidates for parent selection or commercial release. Successful population improvement is necessary to ensure that the product development pipeline remains competitive. Genotype‐by‐environment interaction (GEI) and the need to improve multiple traits are two factors that can limit a breeding program's success if not carefully addressed.

Differential responses of genotypes to changes in their environment result in GEI. Breeders often conduct multi‐environment trials (METs) and fit multivariate linear mixed models to evaluate breeding candidates’ performance across the target population of environments (TPE). Single‐trait multi‐environment (STME) models capture GEI for each trait analyzed separately; however, because traits are modeled independently, they cannot exploit or account for genetic covariances between traits. Integrating multi‐trait multi‐environment (MTME) data further increases model complexity, especially when traits are not uniformly phenotyped across environments, a common occurrence due to cost constraints. As a result, many breeding programs simplify selection models by omitting one or more of these key aspects. Among the various statistical approaches for the analysis of MET data (Bančič et al., 2023; Montesinos‐López et al., 2016, 2019; A. B. Smith & Cullis, 2018; A. Smith et al., 2001; A. B. Smith et al., 2007), the factor analytic (FA) linear mixed model is especially attractive due to its parsimony, flexibility, interpretability, and ability to efficiently capture genetic covariance heterogeneity between environments (A. Smith et al., 2001; A. B. Smith et al., 2007). While Bayesian hierarchical MTME models offer greater flexibility (Montesinos‐López et al., 2016, 2019), they are computationally intensive and scale poorly with large datasets (Angelino et al., 2016; Wu et al., 2012; Zhou et al., 2013). In contrast, FA models strike a favorable balance between model complexity and computational feasibility. Recent advancements have also extended breeders’ selection tools for the FA linear mixed model, providing informative measures of overall performance and stability across traits and environments (Bančič et al., 2023).

Selection methods in breeding programs must not only address GEI but also address the need to improve the population for multiple traits. This can be especially challenging when traits are genetically and unfavorably correlated because selection for one trait will lead to a correlated response to selection in another trait due to pleiotropy or linkage disequilibrium (Falconer & Mackay, 1996). Traditionally, procedures such as tandem selection and independent culling levels have been used for multi‐trait selection, but these methods are suboptimal for parent selection because they do not account for genetic correlations between traits and because extra merit in one trait will not compensate for a lack of merit in another. For optimal multi‐trait selection, the Smith–Hazel index (SHI) (Hazel, 1943; H. F. Smith, 1936) is available. In its original form, the SHI linearly combines phenotypic data on multiple traits using optimal weights computed using economic weights along with genetic and phenotypic covariances to maximize gain in total net merit. Net merit denotes the aggregate economic breeding value of a genotype, which is its expected overall economic return across all traits given the defined economic weights. Henderson (1963) showed that predictions of breeding value using all phenotypic information in best linear unbiased prediction can be directly weighted by economic value (EV) to compute a SHI (Hazel et al., 1994). Subsequent extensions to genomic selection have adopted a similar approach by considering genomic predictions of breeding value (e.g., Ceron‐Rojas et al., 2015; Dekkers, 2007). For a given trait, the economic weight quantifies how much a one‐unit change in trait value impacts the total profit earned by the producer in monetary units per acre, holding all other traits constant. Economic weights are specific to a breeding program's TPE and market segments. To estimate economic weights for multiple traits, profit functions (Moav & Moav, 1966) and other methodological approaches can be used, as reviewed by Nielsen et al. (2014). It is important to emphasize that linear selection indices are optimized to maximize gain in the next generation; thus, these methods are directly applicable to parent selection within breeding programs, whereas selection for product development requires different methods.

In animal breeding, economic indices have long been used to achieve genetic gain across multiple traits. Successful applications have been reported in cattle (*Bos tauru*s) with weight traits (Fernandes et al., 2018) and the interval between first and second calving (Amaya et al., 2020). Examples in pigs (*Sus scrofa*) include meat quality traits with intermediate optima (Hovenier et al., 1993) as well as reproductive, growth, and carcass traits (Dube et al., 2013). In contrast, most crop breeding programs rely on heuristic methods for multi‐trait selection, or they may employ weights assigned based on the perceived importance of each trait due to a lack of research on the EVs of traits of interest in the regions of interest. Unlike in animal breeding, the economic importance of traits in crop breeding is expected to vary widely across and even within regions due to the diversity of crop production systems, markets, and environmental conditions found within a breeding program's TPE. When explicit EVs are unavailable, or unreliable, the desired gains index provides a practical alternative by solving for index coefficients that deliver user‐specified proportional responses across traits (Pešek & Baker, 1969). Recent work formalizes its connection to the Smith–Hazel solution (Werner et al., 2024) and demonstrates balanced responses under price uncertainty in animal breeding (Sánchez et al., 2021), with maize (*Zea mays* L.) studies similarly moving toward indices that reduce reliance on ill‐defined weights (Olivoto et al., 2021). Although the desired gains index provides a good solution for multi‐trait selection, in some cases, economic weights can be readily obtained. In these instances, determining and utilizing empirically derived economic weights would guide selection decisions toward farmer and industry priorities.

Soft red winter wheat (SRWW; *Triticum aestivum* L.) is an important cereal crop in the central US Midwest, including Illinois, where it is commonly grown as part of a wheat–soybean (*Glycine max* L.) double‐crop system. In this system, wheat is planted from late September to early October, harvested in late June to early July, and soybean is planted immediately afterward. Grain yield (GY) of both wheat and soybean is a primary determinant of crop revenue because it directly influences gross returns per unit area. Timely wheat maturity (MAT) is critical because delayed soybean planting shortens the soybean growing season, reduces vegetative and reproductive development, and ultimately decreases yield potential (Hu & Wiatrak, 2012). Several studies in the US Midwest have reported soybean yield penalties when planting is delayed beyond the optimal window (Bastidas et al., 2008; De Bruin & Pedersen, 2008; Egli & Cornelius, 2009; Loman et al., 2024; Pedersen & Lauer, 2004; Rowntree et al., 2013). In wheat breeding programs, heading time (HD) is often used as a proxy for maturity date because the two traits are highly correlated and because HD can be evaluated more quickly and accurately.

In addition to GY and MAT, test weight (TW) is a key quality trait in wheat breeding. TW is defined as the weight of grain that fills a standardized volume and serves as a rapid, indirect measure of grain bulk density and soundness. High TW values are generally associated with plump kernels, superior milling properties, and higher flour extraction rates. Low TW can result from Fusarium head blight (FHB) caused by *Fusarium graminearum*, poor grain development, or pre‐harvest sprouting. FHB infection can also lead to the accumulation of deoxynivalenol (DON), a mycotoxin that poses food safety risks and triggers additional price discounts or load rejection at grain elevators when concentrations exceed market thresholds. In the US grain marketing system, TW is a federally recognized grading factor, and most grain elevators enforce discount schedules when TW falls below a threshold (U.S. Department of Agriculture, Agricultural Marketing Service [USDA‐AMS], 2014). A “discount” refers to a price deduction applied per unit of grain sold when quality specifications are not met. These deductions are proportional to the deviation from the standard threshold, with steeper penalties for more severe deficiencies. In some cases, loads with extremely low TW may be rejected outright due to processing inefficiency or poor end‐use quality. For producers, discounts can represent substantial economic losses, as they directly reduce the final sale price received for the crop. Consequently, maintaining adequate TW is a critical breeding objective, both to meet federal grade requirements and to protect farmer profitability.

Munaro et al. (2025) evaluated 21 years of SRWW breeding progress at the University of Illinois and found that genetic trends for GY, TW, and HD were favorable, but due to the influence of the environment, genetic trends in GY were not enough to generate favorable phenotypic trends. Greater rates of genetic gain would be needed to ensure that the breeding program has a visible impact. Given the complexity of multi‐trait selection in this environment, a genomic‐based SHI that incorporates a single‐step MTME model with empirically derived economic weights has the potential to increase rates of genetic gain and enhance breeding efficiency. With this goal in mind, the objectives of this research are to (i) implement a single‐step MTME model and SHI to rank genotypes for parent selection based on total net merit; (ii) confirm the superiority of this approach compared to the standard STME analysis; (iii) assess the accuracy of MTME genomic estimated breeding values (GEBVs); and (iv) explore genetic correlations among traits and environments.

## 2 MATERIALS AND METHODS

### 2.1 Phenotypic data and experimental design

The dataset comprises 38,792 phenotypic observations collected from the SRWW yield trials at the University of Illinois wheat breeding program over 3 years (2022–2024), across four Illinois locations: Addieville (Adv), Neoga (Neo), St. Peter (Stp), and Urbana (Urb) (Figure 1). This results in a total of 12 different environments, each defined by a unique year–location combination.

**FIGURE 1:**
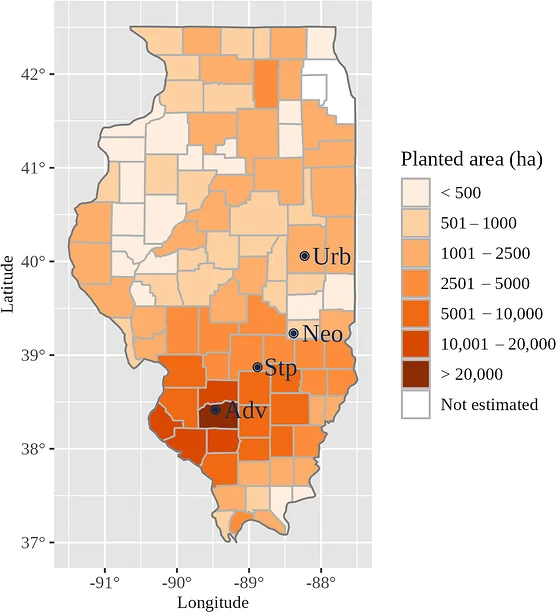
Map of Illinois showing the four test locations included in this study, Addieville (Adv), Neoga (Neo), St. Peter (Stp), and Urbana (Urb). The orange shades represent the 2024 wheat planted hectares (ha) per county (U.S. Department of Agriculture, Farm Service Agency [USDA‐FSA], 2024).

Each trial employed a partially replicated design, ensuring at least 20% replication, with replicated genotypes being tested twice, in a rectangular row‐column arrangement (Table 1). A sparse testing strategy was used across environments, where genotypes from all stages of selection were randomized together to avoid genotype and environment confounding (Ackerman & Rutkoski, 2026). Genotypes in the early stages of selection were evaluated in fewer locations and with fewer replications due to the large number of individuals and the limited seed availability at this stage. In contrast, more advanced genotypes were evaluated in more environments and served as replicates, in addition to commercial checks. Each year, approximately 30% of lines were selected for continued evaluation in subsequent trials. The number of genotypes assessed per environment varied from 486 to 1337 (Table 1), while the number of shared genotypes between environments ranged from 35 to 855 (Figure 2).

**TABLE 1:** Summary of the number of genotypes, plots, and trait data records by growing environment.

Env	Gen			GY (t ha^−1^)		TW (g L^−1^)		HD (JD)		HT (cm)		MAT (JD)	
	Total	Rep (%)	Plots	Mean	NA	Mean	NA	Mean	NA	Mean	NA	Mean	NA
22‐Adv	486	41	684	4.8	1	750	1						
22‐Neo	597	41	840	6.8	0	789	0	133	0	101	0		
22‐Stp	486	37	666	6.2	0	790	0						
22‐Urb	1337	20	1598	6.8	2	795	2	134	0	100	0		
23‐Adv	507	33	676	6.6	0	771	0						
23‐Neo	860	20	1032	8.4	0	800	0	128	0	107	6		
23‐Stp	508	35	684	6.1	10	824	10			88	10		
23‐Urb	1037	25	1296	6.9	0	782	0	131	0	101	0	166	0
24‐Adv	540	20	650	5.9	1	721	1						
24‐Neo	858	20	1032	6.9	6	721	7	119	2	99	0		
24‐Stp	513	23	630	6.5	0	731	1						
24‐Urb	927	22	1128	5.3	2	711	50	124	1	101	1	164	1
Overall	2995	28	38,792	6.5	22	767	72	129	3	100	17	165	1

*Note*: Blank cells indicate that the trait was not measured in the environment. Environments are defined as the combination of year and location. Locations include Addieville (Adv), Neoga (Neo), St. Peter (Stp), and Urbana (Urb).

Abbreviations: Env, environment; Gen, genotype; GY, grain yield; HD, heading time; HT, height; JD, Julian date; MAT, maturity; NA, missing data; Rep (%), replication percentage; TW, test weight.

**FIGURE 2:**
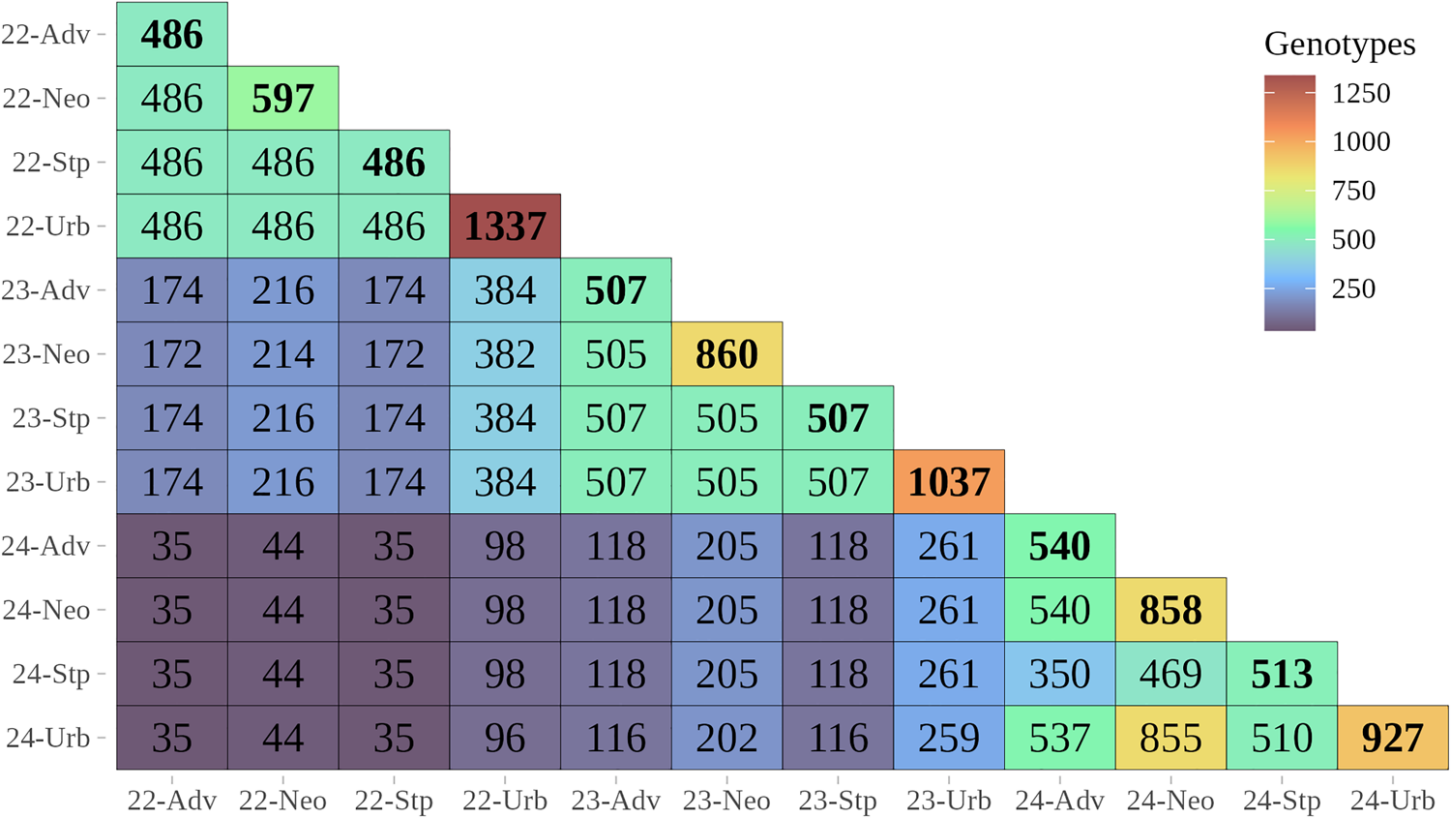
Connectivity in the dataset based on the number of genotypes in common between pairs of environments.

Five traits were measured: GY, TW, HD, plant height (HT), and maturity (MAT). GY and TW were measured on whole plots using a HarvestMaster H2 weigh system (Juniper Systems). GY was then standardized to 13.5% moisture. The HD of each plot was determined by assessing each plot on alternating days and recording the Julian date when at least 50% of the wheat heads had emerged. HT was evaluated as an overall average plant height across entire plots using a measuring stick. Likewise, for MAT, plots were assessed on alternating dates to determine when at least 50% of the peduncles and wheat heads had lost all green color. GY and TW were recorded across all environments. HD, HT, and MAT were assessed in six, seven, and two environments, respectively (Table 1). The phenotypes for each trait were then scaled to unit variance for model fitting, with model parameters transformed back to their original scale after estimation.

### 2.2 Marker data

For each genotype, desiccated leaf‐tissue samples were submitted to the USDA‐ARS Eastern Regional Small Grains Genotyping Lab for genotyping via genotyping‐by‐sequencing (Elshire et al., 2011) using the protocol reported by Poland et al. (2012). The resulting marker data included marker information for 2980 out of the 2995 genotypes, coded as 0, 1, or 2 based on a set of 9588 SNPs with less than 50% total missing data. Missing marker data were imputed with Beagle 5.4 using default parameters (Browning et al., 2018).

Markers were filtered using the *ASRgenomics* package in R (Gezan et al., 2022), applying a minor allele frequency threshold >5%, which retained 9210 markers for analysis. To estimate genetic relationships among the genotypes, the genomic relationship matrix, G, was constructed using the first VanRaden (2008) method. Three potential duplicate genotypes with pairwise kinship values greater than two were identified and subsequently removed to avoid singularities during model fitting. To ensure positive definiteness and prevent computational issues, the G matrix was adjusted using the *G.tuneup* function. This “bending” process modifies the matrix's eigenvalues, improving its numerical stability. Following this adjustment, the G matrix was inverted to obtain the inverse matrix, $G^{-1}$, using the *G.inverse* function.

### 2.3 Economic values

The EV of a wheat genotype in a double‐crop soybean system, expressed in $ ha^−1^, can be formulated as:

(1) $$\mathrm{EV}\;=\;\mathrm{GY}\times P_{w}-\Delta Y\times P_{s}$$

where GY is the wheat grain yield (t ha^−1^); $P_{w}$ is the wheat price ($ t^−1^); ΔY is the soybean yield penalty due to delayed planting (t ha^−1^); and $P_{s}$ is the soybean price ($ t^−1^). The wheat price ($P_{w}$) is the market price ($P_{\mathrm{wm}}$) minus potential discounts (D) applied at the elevator:

$$\;P_{w}=P_{\mathrm{wm}}\;-D$$

whereD is a function of test weight (TW) in g L^−1^ and the discount rate per unit deviation (d) from the industry standard threshold, TW_0_. Assuming no other discounts are trait‐dependent:

$$D=\left\{\begin{array}{c} \left(TW_{0}-\;\mathrm{TW}\right)\times d,\;TW\leq TW_{0}\\ 0,\;TW>TW_{0} \end{array}\right.\;.$$

Therefore, *D* > 0 occurs when TW is less than TW_0_. To derive a linear weight for TW, we assumed TW ranges from 747 to 682 g L^−^^1^ where TW_0_ **= 747 and 682 g L^−^^1^ is a common threshold for rejection. Total phenotypic values in TW in Illinois wheat breeding trials have been trending downward at a rate of −2.39 ± 0.77 g L^−1^ year^−1^ driven by nongenetic causes (Munaro et al., 2025), supporting our assumption that TW below the industry standard threshold is relevant when estimating its economic weight. Substituting into Equation (1) gives:

$$\mathrm{EV}\;=\;\mathrm{GY}\times \left[P_{\mathrm{wm}}-\left(TW_{0}-\;\mathrm{TW}\right)\times d\right]-\Delta Y\times P_{s}.$$

The ΔY is estimated from the delay in planting due to later wheat maturity. If HD is used as a proxy for maturity:

$$\Delta Y=\Delta \mathrm{HD}\times p_{d}$$

where ΔHD is the difference in heading time (days) between a given genotype and the earliest known genotype, and $p_{d}$ is the soybean yield loss per day of delay (t ha^−1^ per day). Given that the HD of the earliest known genotype is a constant value, HD can be substituted for ΔHD without affecting selection. Thus:

$$\mathrm{EV}\;=\;\mathrm{GY}\times \left[P_{\mathrm{wm}}-\left(TW_{0}-\;\mathrm{TW}\right)\times d\right]-\left(\mathrm{HD}\times p_{d}\right)\times P_{s}.$$

This equation shows that EV is a linear function of GY, TW, and HD. Therefore, the economic weights (w) for these traits are the partial derivatives of EV with respect to each trait, holding the others constant:

$$\;w_{\mathrm{GY}}=\frac{\partial \mathrm{EV}}{\partial \mathrm{GY}}=P_{\mathrm{wm}}\;,$$

$$\;w_{\mathrm{TW}}=\frac{\partial \mathrm{EV}}{\partial \mathrm{TW}}=\mathrm{GY}\times d,$$

$$\;w_{\mathrm{HD}}=\frac{\partial \mathrm{EV}}{\partial \mathrm{HD}}=-p_{d}\times P_{s},$$

GY is expressed in t ha^−1^ and the discount slope (d) is in $ t^−1^ (g L^−1^)^−1^. In order to obtain an economic weight for TW that is independent of the genetic value for yield, $w_{\mathrm{TW}}$ can be solved by substituting GY with the average yield ($\bar{\mathrm{GY}}$) of the state or region of interest:

$$\;w_{\mathrm{TW}}=\bar{\mathrm{GY}}\;\times d.$$

Similarly, $w_{\mathrm{HD}}$ can be solved assuming $p_{d}=0.0336$ t ha^−1^ day^−1^, supported by unpublished research in Illinois conducted between 2010 and 2015 (Emerson Nafziger, personal communication, 2019), and $P_{s}$ equal to the 10‐year average soybean price (${\bar{P}}_{s}$):

$$\;w_{\mathrm{HD}}=-0.0336\times {\bar{P}}_{s}.$$

The empirical values used in this study included the monthly average wheat and soybean prices over the past 10 years (2015–2024) from U.S. Department of Agriculture, National Agricultural Statistics Service (USDA‐NASS) (2025) (Figure 3). To quantify the economic impact of TW variations, we reviewed discount schedules from seven grain elevators located in Illinois (Figure 4).

**FIGURE 3:**
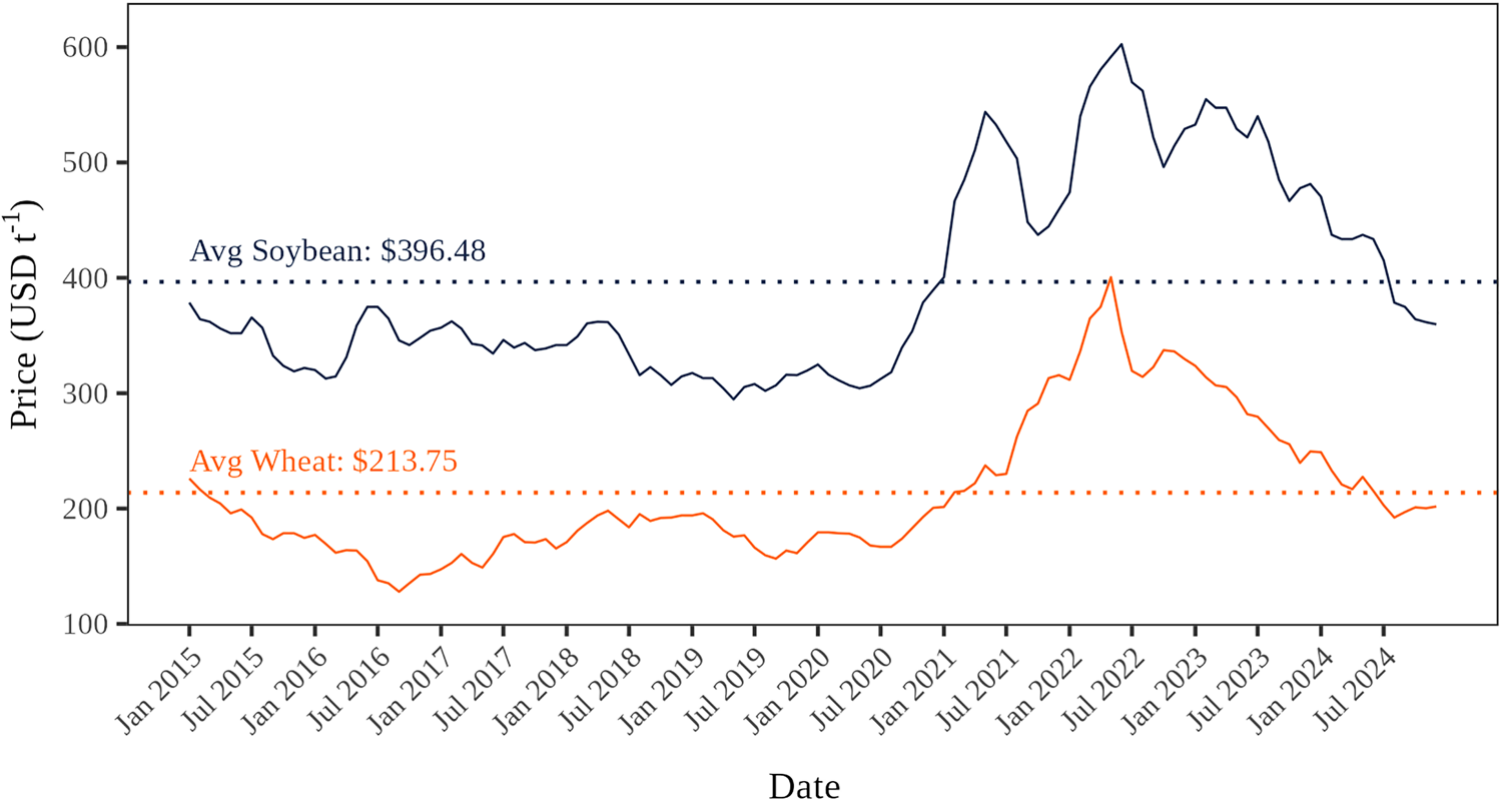
Monthly wheat and soybean prices over a 10‐year period (2015–2024). The histogram on the right illustrates the distribution of the wheat‐to‐soybean price ratio. The dashed horizontal lines represent the average wheat and soybean prices over the period in $ t^−1^. Although both wheat and soybean prices have fluctuated over the past decade, the price ratio between these crops has remained relatively stable.

**FIGURE 4:**
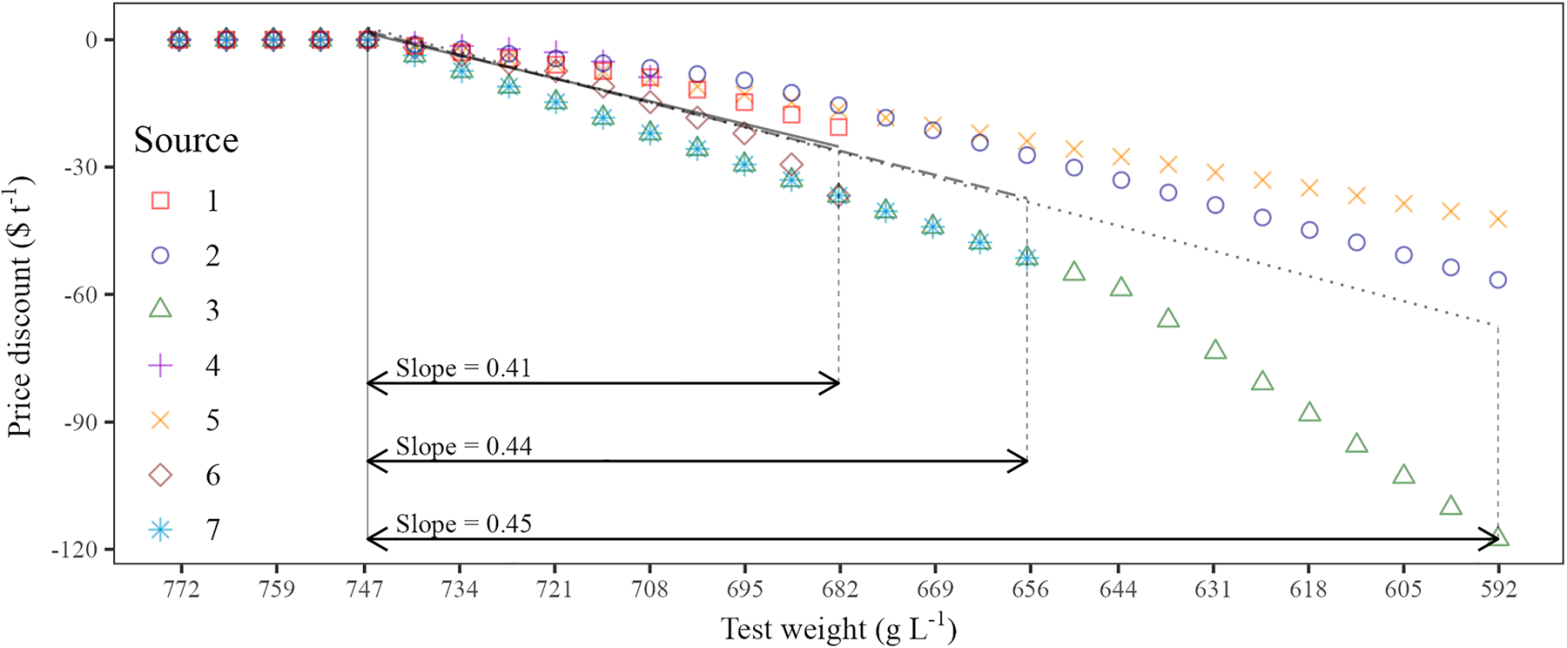
Discounts applied to the wheat market value based on test weight, retrieved from seven different sources. The solid vertical line represents the test weight threshold at which discounts begin, while the dashed vertical lines indicate the test weight values at which grain may be subject to rejection, depending on the discount schedule source. Regression lines illustrate the discount progression for each interval between the initiation of discounts and the rejection threshold, with corresponding slopes indicated. The *y*‐axis progresses from negative values toward zero; therefore, the slopes are positive. Slopes are in units of $ t^−1^ (g L^−1^)^−1^.

By regressing discount rates from multiple sources, we established an estimated discount slope ($\hat{d}$) that reflects regional industry practices. Substituting empirical values from this study:

$$\;{\bar{P}}_{\mathrm{wm}}=213.75\;\$\;t^{-1},$$

$$\;\bar{\mathrm{GY}}=6.5\;t\;ha^{-1},$$

$$\;\hat{d}=0.41\;\$\;t^{-1}{\left(gL^{-1}\right)}^{-1},$$

$$\;{\bar{P}}_{s}=396.43\;\$\;t^{-1},$$

$$\;p_{d}=0.0336\;t\;ha^{-1}\mathrm{da}y^{-1},$$

Thus, the final vector of economic weights for GY, TW, and HD is:

$$w={\left[213.75,\;2.665,\;-13.32\right]}^{⊤}$$

All other traits had a weight of zero. Although DON is another important trait impacting profitability, the current study does not incorporate this trait in the index.

### 2.4 Statistical models

The multi‐trait MET dataset comprised $n_{t}$ = 5 traits and $n_{e}$ = 12 environments with $n_{te}=39$ observed trait–environment combinations and $n_{g}$ = 2977 genotypes with $n_{m}$ = 9210 markers for n = 38,792 plots in total. The traits were recorded in at least two environments, with yield and TW recorded in all 12 environments. The environments contained field trials laid out as a rectangular array comprising $c_{j}$ columns and $r_{j}$ rows, with $n_{j}=c_{j}\;r_{j}$ plots in total. A partially replicated design was used for each trial following Cullis et al. (2006), with replication ranging from 20% to 41% (Table 1). Missing phenotypes were assigned a missing value indicator in the data to maintain the spatial layout required for subsequent model fitting. Finally, let the n‐vector of scaled phenotypic data be given by $y={\left(y_{1}^{⊤},\;\text{…},\;y_{s}^{⊤}\right)}^{⊤}$, where $y_{i}$ is the $n_{i}$‐vector for the ith trait across environments. The combination of trait i and environment j is hereafter referred to as the hth trait–environment combination, such that the length of y is $n=\sum_{h=1}^{n_{te}}n_{h}$. The linear mixed model for y can be written as:

(2) y=Xτ+Zu+Wb+e

where τ is a vector of fixed effects with design matrix X; u is a vector of random genotype effects for each trait–environment combination with associated design matrix Z, and will be referred to as the genotype‐by‐environment‐by‐trait (GET) effects, b is a vector of random block effects with design matrix W, e is the vector of residuals, including spatial modeling (Gilmour et al., 1997). The effects in τ include the mean parameter for each trait by environment combination and fixed effects for the 15 genotypes without marker data (i.e., check cultivars or entries without DNA samples). While these genotypes were of no interest to the breeding objective, their phenotypic information was retained to improve spatial modeling (Tolhurst et al., 2019). The effects in b include experimental design terms capturing the blocking structure of trials. All random terms were assumed to be mutually independent following a multivariate normal distribution with mean zero and variance structures defined below.

#### 2.4.1 Variance model for the nongenetic effects and residuals

The block variance structure was assumed to be diagonal as $\mathrm{var}\left(b\right)={⊕}_{i=1}^{n_{te}}\;\sigma_{b_{h}}^{2}I_{n_{b}}$, with $n_{b}=2$ blocks for each trait–environment combination and $n_{te}=39$ variance components in total. The residuals for each trait–environment combination were assumed to be independent with their own spatial model. While this approach does not model the residual correlations between traits (De Faveri et al., 2017), we found that it provides a practical and computationally efficient variance structure for the residuals. Thus, the residual variance structure was assumed to be block diagonal as $\mathrm{var}\;\left(e\right)={⊕}_{h=1}^{n_{te}}\;R_{h}$, where $R_{h}=\sigma_{h}^{2}\;\Sigma_{c_{h}}\left({\rho_{c}}_{h}\right)⊗\Sigma_{r_{h}}\left({\rho_{r}}_{h}\right)$ is the spatial model for the hth trait–environment combination, resulting in $3n_{te}$ variance and correlation parameters in total (A. B. Smith et al., 2007). The spatial model for each combination was chosen as a separable two‐dimensional autoregressive process of order one (Cullis & Gleeson, 1991), with separate autocorrelation parameters fitted to the columns and rows, ${\rho_{c}}_{h}$ and ${\rho_{r}}_{h}$, respecting the experimental design and spatial layout of trials.

#### 2.4.2 Variance models for the genetic effects

The genetic variance matrix was assumed to be $\mathrm{var}\;\left(u\right)=G_{\mathrm{te}}\;⊗G$, where $G_{\mathrm{te}}$ is a $n_{\mathrm{te}}\times n_{\mathrm{te}}$ non‐separable matrix between trait–environment combinations and G is a $n_{g}\times n_{g}$ genomic relationship matrix between genotypes (VanRaden, 2008). The variance model for the genetic effects was chosen as the FA model of A. Smith et al. (2001), as applied to MTME data by A. B. Smith et al. (2007), and extended for genomic prediction by Bančič et al. (2023). This model was fitted directly to the individual trait–environment combinations in $G_{\mathrm{te}}$ and is referred to as FA k model, where k denotes the number of common factors. The FA k model can be written as:

(3) $$\begin{array}{ccc} u & = & \left(\lambda_{1}⊗I_{n_{g}}\right)\;f_{1}+\cdots +\left(\lambda_{k}⊗I_{n_{g}}\right)f_{k}+\delta \\ & = & \left(\Lambda ⊗I_{n_{g}}\right)\;f+\delta \; \end{array}$$

where $\Lambda =[\lambda_{1}\text{…}\;\lambda_{k}]$ is a $n_{te}\times k$ matrix of trait–environment loadings, $f={\left(f_{1}^{⊤},\text{…},f_{k}^{⊤}\right)}^{⊤}$ is a $n_{g}k$‐vector of genotype scores, and $\delta ={\left(\delta_{1}^{⊤},\text{…},\delta_{n_{te}}^{⊤}\right)}^{⊤}$ is a $\;n_{g}n_{te}$ ‐vector of regression residuals that are specific to individual trait–environment combinations.

Following A. Smith et al. (2021), the loadings are assumed to have orthonormal columns, such that $\Lambda^{T}\Lambda =I_{k}$. It is also assumed that:

$$\left[\begin{array}{c} f\\ \delta \end{array}\right]∼N\left(\left[\begin{array}{c} 0\\ 0 \end{array}\right],\left[\begin{array}{cc} D & 0\\ 0 & \Psi \end{array}\right]⊗G\right)$$

where $D={⊕}_{r=1}^{k}\;d_{r}$ is a diagonal matrix with score variances ordered as $d_{1}>\text{…}>d_{k}$ and $\Psi ={⊕}_{h=1}^{n_{te}}\;\psi_{h}$ is a diagonal matrix with specific variances for individual trait by environment combinations denoted by $\psi_{h}$. The variance matrix for u is therefore given by:

(4) $$\mathrm{var}\;\left(u\right)=\left(\Lambda D\Lambda^{⊤}+\Psi \right)\;⊗G.$$

#### 2.4.3 Model estimation and selection

All linear mixed models were fitted in ASReml‐R (Butler et al., 2023), which obtains residual maximum likelihood (REML) estimates of the variance parameters. The FA models included separate terms for the latent regression given by $\beta \;=(\Lambda ⊗I_{v})\;f$ and the regression residuals given by δ, fitted in ASReml‐R using reduced rank and diagonal variance structures. The associated variance parameters are the loadings, score variances, and specific variances, and the REML estimates of these parameters will be denoted by ${\hat{\lambda }}_{rh},\;{\hat{d}}_{r}$ and ${\hat{\psi }}_{h}$. Genomic best linear unbiased predictions (GBLUPs) were obtained as solutions to the mixed model equations, with GBLUPs of the key genetic effects denoted by $\tilde{\beta }$, $\tilde{\delta }$, and $\tilde{f}$. Following A. B. Smith and Cullis (2018), GBLUPs of the total GET effects were obtained as:

(5) $$\tilde{u}=\tilde{\beta }+\tilde{\delta }.$$

Following A. B. Smith and Cullis (2018), the REML estimates of the trait–environment loadings and genotype scores were rotated to a principal component solution after estimation, with the first rotated factor accounting for the maximum amount of covariance in the GET effects, the second accounting for the next largest amount and being orthogonal to the first, and so on. After rotation, the percentage of additive genetic variance explained by factor *r* for the hth trait–environment combination was computed as:

(6) $$v_{rh}=100\frac{{\left({\hat{\lambda }}_{rh}\right)}^{2}{\hat{d}}_{r}}{\mathrm{VA}R_{h}}$$

where $\mathrm{VA}\;R_{h}=\sum_{r=1}^{k}{\left({\hat{\lambda }}_{rh}\right)}^{2}{\hat{d}}_{r}+{\hat{\psi }}_{h}$ is the total genetic variance. In this way the mean contribution of factor r to the additive genetic variance can be computed across all trait–environment combinations as $v_{r}=\sum_{h=1}^{n_{te}}\frac{v_{rh}}{n_{te}}$ and overall as ${\bar{V}}_{a}=\sum_{r=1}^{k}\frac{v_{r}}{k}$.

The model‐based accuracy for the total GET effect of genotype *i* for the *h*th trait–environment combination was computed following A. B. Smith and Cullis (2018) as:

(7) $$r_{ih}=\sqrt{\;1-\frac{\mathrm{PE}V_{ih}}{\mathrm{VA}R_{ih}}}\;$$

where $\mathrm{PE}V_{ih}$ is the prediction error variance, obtained by summing contributions from the latent regression and regression residuals, and $\mathrm{VA}R_{ih}$ is the corresponding genetic variance, computed as $\mathrm{VA}R_{h}$ multiplied by the *i*th diagonal element of the genomic relationship matrix.

#### 2.4.4 Selection index

We employed a genomic‐based SHI to compute the net merit of each genotype, which is given by:

(8) $$\mathrm{SHI}\;=w^{T}\;\tilde{u}$$

where **w** is the vector of economic weights derived in Section 2.3 and $\tilde{u}$ are the GBLUPs of the genotype by trait effects, obtained by averaging over environments. This assumes that each environment is equally representative of our TPE, which is what we expect.

### 2.5 MTME versus STME

The multi‐trait MET dataset was analyzed using two linear mixed models. First, the MTME model, implemented as described in Section 2.4, incorporates all traits simultaneously. Then, the STME model was applied, where each trait was analyzed independently using the same model structure but assuming independence across traits. Under the STME model, the SHI was computed by applying the same vector of economic weights to GBLUPs obtained from the independent single‐trait analyses. That is, GBLUPs for GY, TW, and HD were extracted from their respective STME models and combined post‐hoc using the economic weights to compute a SHI for each genotype. Therefore, the genetic variance matrix for STME model is given by $\mathrm{var}\left(u\right)=I_{n_{t}}\;⊗G_{e}⊗G$, where $I_{n_{t}}$ is a $n_{t}\times n_{t}$ diagonal matrix between traits, $G_{e}$ is a $n_{e}\times n_{e}$ variance matrix between environments, and G is as previously defined.

#### 2.5.1 Response to selection

To evaluate the impact of different models and selection criteria, where selection criteria refer to selecting the top genotypes based on their average SHI or GY across all environments, we compared the MTME‐predicted values for SHI and other traits of the selected individuals (top 10%) to those of the full candidate population. Additionally, we identified the top 10% of individuals using the STME model and calculated the expected response to selection relative to MTME. The response to selection was quantified as the difference between the mean performance of the top 10% selected genotypes and the mean performance of all selection candidates, excluding genotypes discarded prior to 2024.

### 2.6 Software, code, and data availability

All data manipulation and analysis were conducted in the R Environment version 4.4.2 (R Core Team, 2024) using the R packages *Tidyverse* (Wickham et al., 2019) for data manipulation, *ASRgenomics* to create the genomic relationship matrix (Gezan et al., 2022), and ASReml‐R (Butler et al., 2023) for data analysis. The R scripts used to perform the analyses are available in the GitHub repository *mtme‐gsi4wheat* (https://github.com/lbmunaro/mtme‐gsi4wheat).

## 3 RESULTS

### 3.1 Model selection and accuracy

A series of factor analytic model with *k* factors (Fa*k*) models (k=1,…,9) were compared to determine the optimal balance between model complexity, proportion of additive genetic variance explained, and computational cost (Table 2; Figure 5). Across all traits, the proportion of additive genetic variance explained (${\bar{V}}_{a}$ *%*) increased from *k* = 1 to *k* = 9, with diminishing returns for *k* > 5 (Figure 5). GY exhibited the largest gain in mean $V_{a}$ *%*, rising from 18% (FA1) to 87% (FA5) and plateauing thereafter. HD was well captured even at low rank, with >96% of $V_{a}$ explained at *k* ≥ 1. HT and TW required moderate factor complexity (*k* ≥ 3 and *k* ≥ 2, respectively) to exceed 90%$\;V_{a}$, whereas MAT remained moderately explained (76%–80% $V_{a}$) across all *k*. Model fit statistics further supported *k* = 5 as a pragmatic choice while ensuring all trait–environment combinations had at least 60% explained for yield (Table 2). Log‐likelihood values increased, and Akaike information criterion values decreased with increasing *k*, but the rate of improvement diminished, and computational time increased substantially beyond FA5. Therefore, FA5, capturing >91% of additive variance on average while maintaining manageable runtime (∼53 h), was selected for all subsequent analyses.

**TABLE 2:** Fit statistics and variance explained for each factor analytic model with *k* factors (FA*k*).

Model	Par	Loglik	AIC	${\bar{V}}_{a}$ (%)	Time (h)
FA1	273	15,645.4	−30,744.7	29.9	3.6
FA2	331	17,529.7	−34,437.4	63.8	3.8
FA3	348	18,303.4	−35,910.8	78.6	18.7
FA4	384	18,768.8	−36,769.6	89.4	38.2
FA5	419	19,095.7	−37,353.5	91.9	53.2
FA6	453	19,230.4	−37,554.8	93.2	73.1
FA7	486	19,340.6	−37,709.1	93.6	91.3
FA8	518	19,404.1	−37,772.2	94.5	120.4
FA9	549	19,443.6	−37,789.1	95.2	122.5

Abbreviations: AIC, Akaike information criterion; Par, number of variance parameters; LogLik, residual maximum likelihood; ${\bar{V}}_{a}$ (%), mean percentage of additive genetic variance explained; Time, computation time in hours (h).

**FIGURE 5:**
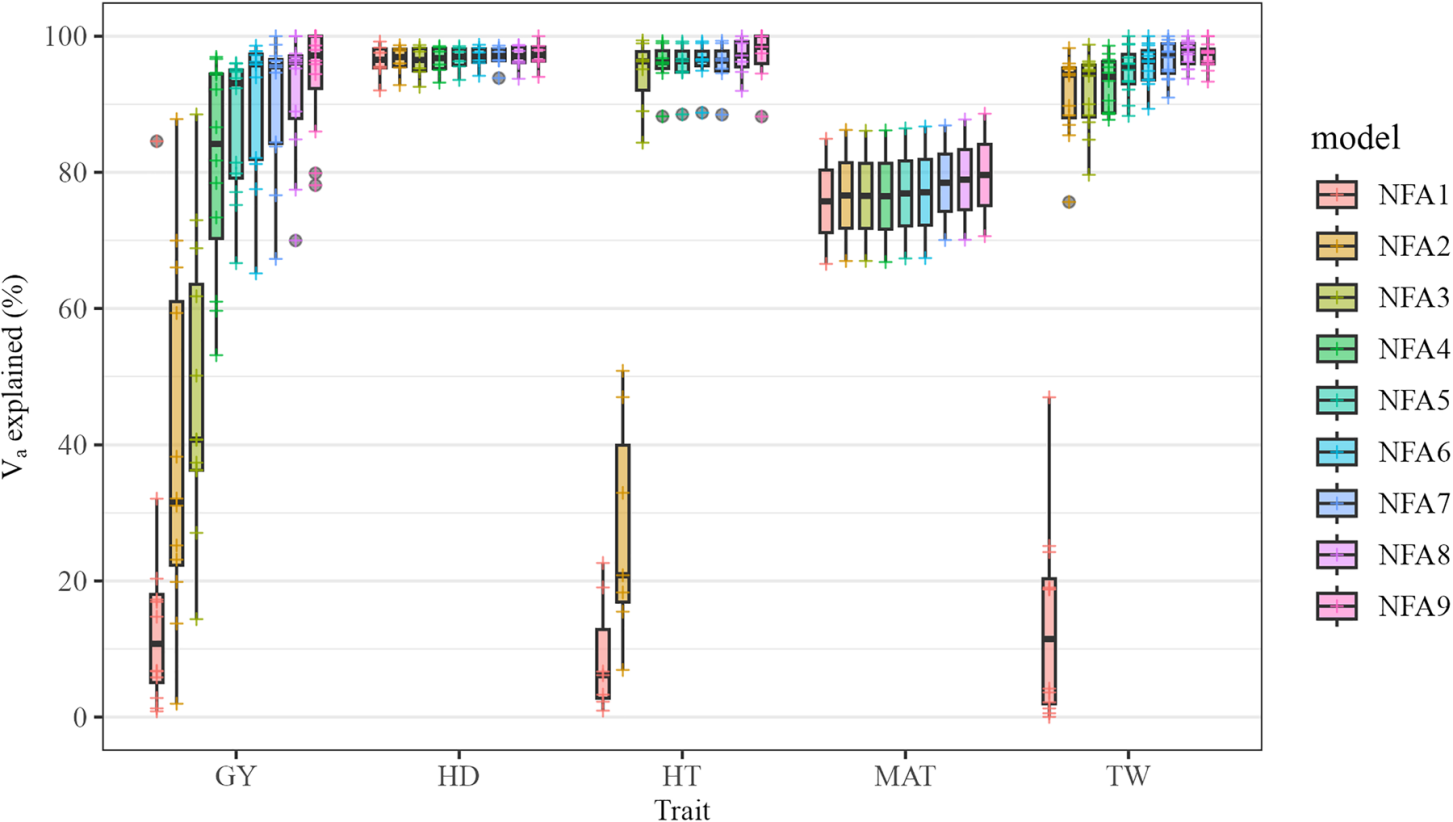
Percentage of additive genetic variance ($V_{a}$) explained by nine factor analytic (factor analytic model with *k* factors [Fa*k*]) multi‐trait multi‐environment genomic selection models for five agronomic traits. Each boxplot represents the distribution of $V_{a}$ explained across all trait–environment combinations for a given trait: grain yield (GY), heading time (HD), plant height (HT), maturity (MAT), and test weight (TW).

The model‐based accuracy of the total GET effects remained uniformly high across all five traits (Figure 6). Across 116,103 GET effects, the mean accuracy was 0.95 for GY, 0.97 for TW, 0.98 for HD, 0.97 for HT, and 0.96 for MAT. HD exhibited the highest average accuracy and the narrowest spread, whereas GY showed the lowest mean accuracy with a slightly wider spread.

**FIGURE 6:**
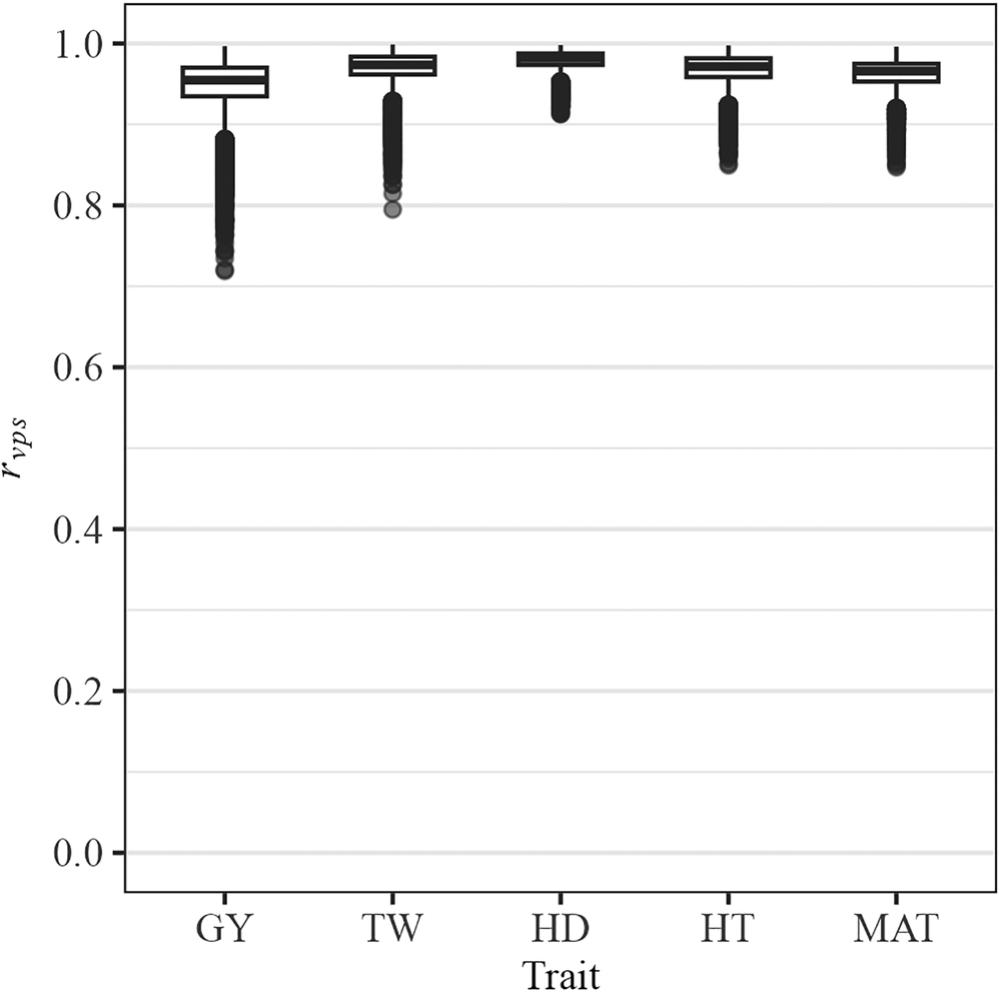
Distribution of model‐based genotype by trait‐environment (GET) effect accuracies (r) for total genetic effects for grain yield (GY), heading time (HD), plant height (HT), maturity (MAT), and test weight (TW).

### 3.2 Correlation among GEBVs from STME and MTME

We assessed selection outcomes based on GEBVs and the SHI predicted using MTME and STME models. Strong linear relationships were observed between MTME and STME predictions for all traits (Figure 7), with Pearson correlation coefficients (*r*) ranging from 0.86 to 0.97. Traits such as HD and HT exhibited the highest correlations (*r* = 0.97), indicating nearly identical predictions from both models. Lower correlations were found for maturity (MAT; *r* = 0.86) and GY (*r* = 0.89), suggesting greater differences between MTME and STME model predictions for these traits, likely reflecting enhanced modeling of GEI and trait correlations in the MTME framework.

**FIGURE 7:**
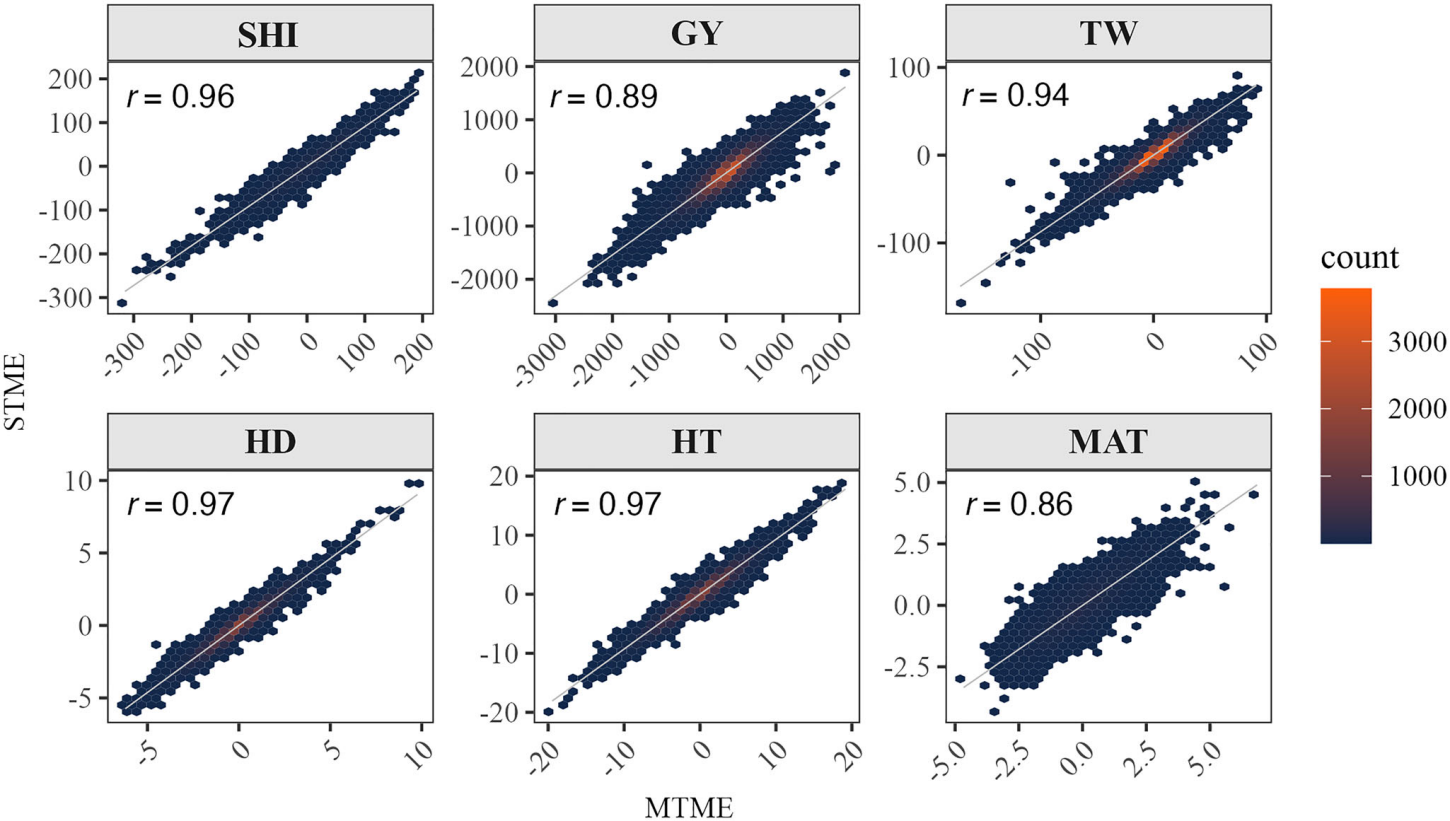
Correlations between single‐trait multi‐environment (STME) and multi‐trait multi‐environment (MTME) genomic estimated breeding values (GEBVs) for grain yield (GY), test weight (TW), heading time (HD), plant height (HT), maturity (MAT), and the genomic‐based Smith–Hazel Index (SHI). The gray line shows the ordinary least‐squares fit, and Pearson's correlation coefficient (*r*) is indicated. Color intensity reflects the number of observations.

### 3.3 Expected response to selection

Selecting the top 10% of genotypes based on either SHI or GY resulted in distinct shifts in the distributions of GEBVs for all evaluated traits (Figure 8). For both selection criteria, the mean SHI and GY of the selected groups exceeded those of the full candidate population. GY‐based selection produced the largest mean increase in GY, whereas SHI‐based selection generated higher gains in TW, with no observable changes in MAT or HD.

**FIGURE 8:**
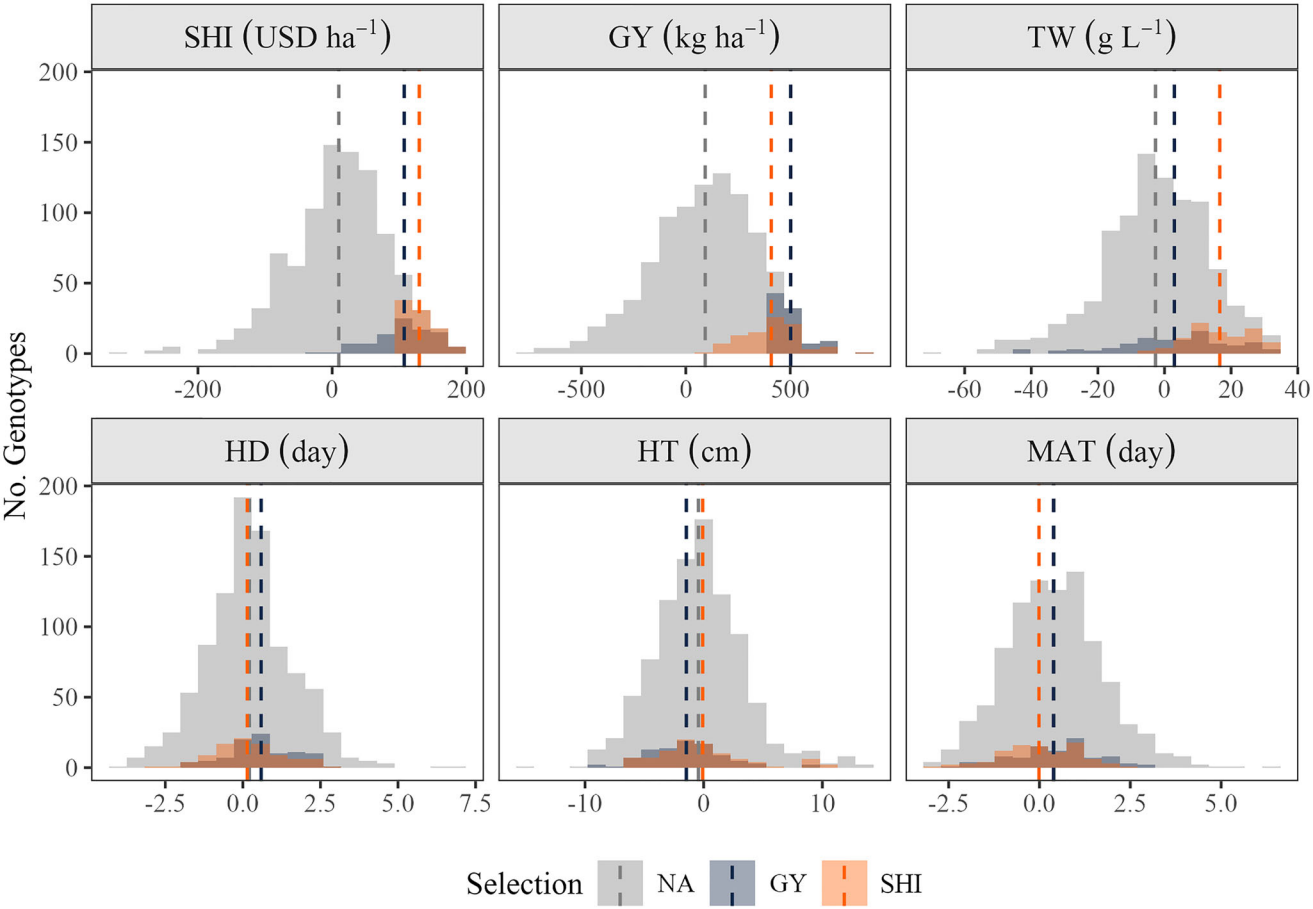
Histograms comparing multi‐trait multi‐environment (MTME)‐predicted genomic estimated breeding values (GEBVs) for full candidate genotypes (gray) and those selected based on grain yield (GY; blue) or the genomic‐based Smith–Hazel Index (SHI; orange). Dashed vertical lines depict the median GEBV for each selection group. HD, heading time; HT, height; MAT, maturity; NA, not applicable; TW, test weight.

Under the MTME model, SHI‐based selection achieved a predicted economic gain of $120 ha^−1^, compared with $116 ha^−1^ for STME‐based SHI selection (Table 3). For GY selection, MTME yielded a gain of 409 kg ha^−1^, exceeding the 390 kg ha^−1^ gain from STME. TW increased by 19.3 g L^−1^ with index selection under MTME and by 18.7 g L^−1^ under STME. In contrast, GY‐based selection resulted in smaller increases in TW: 5.7 g L^−1^ for MTME and 6.0 g L^−1^ for STME. For HD, SHI selection slightly reduced mean values under both MTME (−0.1 day) and STME (−0.3 day), whereas GY selection increased HD by 0.4 days (MTME) and 0.2 days (STME). HT increased by 0.4 cm for MTME and 0.2 cm for STME when selection was based on SHI, while GY‐based selection decreased HT by 1.0 cm for both models. For MAT, the mean changes resulting from index selection were −0.4 days under MTME and −0.6 days under STME, whereas GY selection produced negligible changes (0 days for MTME and −0.3 days for STME).

**TABLE 3:** Predicted response to selection for each trait under multi‐trait multi‐environment (MTME) and single‐trait multi‐environment (STME) genomic prediction models using a genomic‐based Smith–Hazel Index (SHI) and grain yield (GY) as selection criteria.

Trait	SHI		GY	
	MTME	STME	MTME	STME
SHI ($ ha^−1^)	120	116	97.5	96.6
GY (kg ha^−1^)	316	292	409	390
TW (g L^−1^)	19.3	18.7	5.7	6
HD (day)	−0.1	−0.3	0.4	0.2
HT (cm)	0.4	0.2	−1	−1
MAT (day)	−0.4	−0.6	0	−0.3

*Note*: Responses represent mean differences between selected and population‐average genomic estimated breeding values.

Abbreviations: HD, heading time; HT, height; MAT, maturity; TW, test weight.

### 3.4 Genetic correlation among trait–environment combinations

Genetic correlations among trait–environment combinations, estimated from the MTME FA5 model, varied substantially, indicating the presence of both trait‐dependent GEI and environment‐dependent genetic covariances between traits (Figure 9). Within a single trait, genetic correlations across environments were consistently high for HD, MAT, and HT (Figure 9A). TW tended to be highly correlated with itself except for a few environment combinations. In contrast, correlations between GY across environments varied widely, reflecting a stronger GEI effect. While most cross‐environment correlations for GY were positive, exceptions included negative correlations between GY‐24‐Urb and several environments, such as GY‐22‐Stp, GY‐22‐Urb, GY‐23‐Adv, GY‐23‐Neo, GY‐23‐Stp, and GY‐23‐Urb.

**FIGURE 9:**
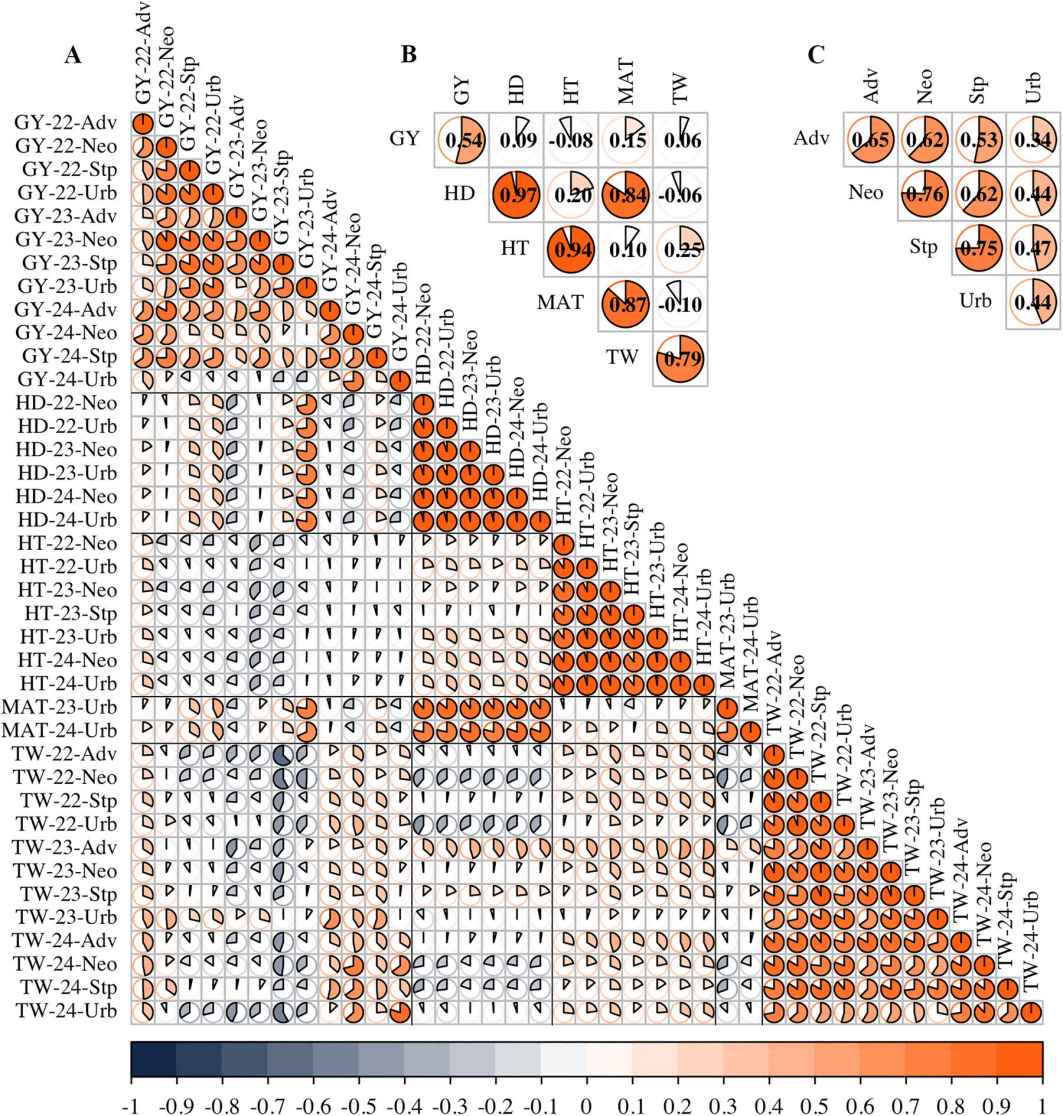
Genetic correlations estimated using the multi‐trait multi‐environment (MTME) factor analytic model with five factors (FA5). Subparts (A) Trait–environment genetic correlation matrix for grain yield (GY), test weight (TW), heading time (HD), plant height (HT), and maturity (MAT) across environments (location‐year combinations: Adv = Addieville, Neo = Neoga, Stp = St. Peter, Urb = Urbana; 22, 23, and 24 denote years 2022, 2023, and 2024). Positive correlations are in orange; negative correlations are in blue. (B) Trait‐level genetic correlation matrix, averaged across environments. (C) Genetic correlations of grain yield across testing locations, averaged across years.

Across traits, correlation values varied considerably, with some trait pairs exhibiting strong positive correlations in specific environments and weak or even negative correlations in others. For instance, HD and MAT were consistently strongly correlated across all environments, while HT showed moderate positive correlations with both HD and TW. In contrast, correlations between GY and other traits were less consistent. For example, GY‐23‐Urb was positively correlated with HD in all environments, whereas GY‐23‐Adv, GY‐24‐Neo, and GY‐24‐Urb showed negative correlations with HD. Similarly, HT generally showed weak or negative correlations with GY, except for GY‐22‐Adv, where the correlation was moderately positive.

Mean within‐trait genetic correlations across environments were moderate for GY (*r *= 0.54), and high for other traits, with correlations ranging from 0.79 (TW) to 0.97 (HD) (Figure 9B). Strong positive correlations were observed between HD and MAT (*r *= 0.84), with moderate associations between HT and HD (*r *= 0.20) and between HT and TW (*r *= 0.25). GY had relatively weak correlations with all other traits, ranging from −0.08 (HT) to 0.15 (MAT) (Figure 9B).

Within GY across locations, average genetic correlations indicated moderate to strong within‐location consistency, with self‐to‐self estimates ranging from *r *= 0.44 (Urb) to *r *= 0.76 (Neo) (Figure 9C). Cross‐location correlations ranged from *r *= 0.34 (Adv vs. Urb) to *r *= 0.62 (Adv vs. Neo and Neo vs. Stp). Neo consistently showed higher genetic correlations both within and across locations, while Urb exhibited weaker correlations.

## 4 DISCUSSION

This study demonstrates the application of a genomic‐based SHI with MTME data and empirically derived economic weights to enhance genetic gain in net merit in a wheat breeding program. The methods we applied address GEI and incorporate EVs of traits to ensure parent selection decisions will improve the total net merit of the breeding population. While this study applies the MTME approach for SRWW breeding in the central US Midwest, it can be applied in other crops and geographies to facilitate population improvement.

The optimal model identified, FA5, provided an efficient compromise between complexity, predictive power, and computational practicality, explaining over 91% of additive genetic variance. This efficiency is critical for practical breeding applications where computational resources and turnaround times can constrain breeding decisions (Bančič et al., 2023; A. B. Smith & Cullis, 2018). Moreover, consistently high accuracies of predicted GET effects across traits validated the robustness and reliability of this modeling approach. The number of common factors required (*k* = 5) to explain most of the additive genetic variance indicates that the genetic covariance structure between trait‐environments is potentially complex (Meyer, 2009).

Predictions from STME and MTME models were highly correlated, indicating strong agreement overall. However, this level of agreement can diminish under stronger GEI or when traits are more highly correlated, conditions under which MTME more efficiently borrows information across traits to improve prediction accuracy (Jia & Jannink, 2012). Direct comparisons of the expected response to selection for the top 10% of genotypes relative to the full candidate population indicated that the MTME‐derived index would lead to higher economic returns than the STME‐derived index if implemented. However, these economic differences were modest, and in situations where computational time is constrained, STME may provide a suitable compromise.

A distinct advantage of MTME models is their capacity to estimate genetic correlations and thus borrow information among trait‐environment combinations, unlike the STME. This is especially beneficial when a trait of low heritability is genetically correlated with another trait of higher heritability (Shahi et al., 2022). Another advantage is the ability to predict performance for all environment‐trait combinations for genotyped lines, even when phenotypic data are missing. This is important when different environments are to be weighed differently. For example, trial sites that are less representative of the TPE can be assigned lower weights, while those that more closely match target conditions can be emphasized in the selection index.

In our case, genetic correlations among economically important traits were generally weak, particularly between GY and other traits, indicating that simultaneous improvement of these traits is readily attainable. Nevertheless, other wheat breeding programs frequently encounter antagonistic correlations. The most notable example is the negative association between GY and grain protein content, which can slow genetic gain when both traits are under selection (Simmonds, 1995; Terman et al., 1969). In such scenarios, MTME models are advantageous because they jointly model trait covariances and facilitate the use of empirically derived economic weights to balance opposing selection pressures and optimize expected economic gain (Bernardo, 2020).

In some cases, the genetic correlations we estimated reflected our firsthand observations of the environmental conditions. For example, HD was consistently positively correlated with GY‐23‐Urb, an environment where a late frost occurred during the boot and early heading stages. Wheat is especially vulnerable to freezing injury during heading and anthesis (Frederiks et al., 2015), and we observed that early‐heading genotypes experienced extensive frost damage and yield loss, leading to a positive correlation between later heading and higher yield. Similarly, in 2024, a severe FHB epidemic occurred in parts of central and southern Illinois, and that year, we observed severe FHB symptoms in our trials, particularly in Urb. This epidemic resulted in strong positive correlations between TW and GY in 2024, particularly in Urb. FHB is known to reduce both GY and TW through kernel damage and mycotoxin contamination (Buerstmayr et al., 2020; McMullen et al., 2012). The reduced correlations between GY‐24‐Urb and other environments in 2022–2023, as well as between TW‐24 and TW in other environments, are also attributable to the severe FHB epidemic we experienced. These examples illustrate how specific biotic and abiotic stresses can modify the expression of genetic variance and covariances among traits, leading to environment‐dependent changes in their apparent genetic architecture (Des Marais et al., 2013; El‐Soda et al., 2014).

While this research represents an important step toward the routine application of economic genomic selection indices in wheat, several limitations should be acknowledged. First, to obtain a single linear economic weight for TW, we assumed an average yield for the TPE and TW below the market standard. Because these assumptions affect the economic weight assigned to TW, this weight would be inaccurate if either assumption is inappropriate. Second, our index assumes no price discount for DON contamination caused by FHB. In practice, DON concentrations exceeding market thresholds lead to price penalties or outright rejection; therefore, DON concentration is an economically important trait that should ultimately be included in our index. Third, lodging tolerance was excluded, although severe lodging is known to cause substantial yield losses, delayed harvest, reduced grain quality, and increased drying costs (Berry & Spink, 2012). Furthermore, if double‐crop soybeans are to be planted, wheat lodging can hinder the establishment of the soybean crop, ultimately reducing its yield. Similarly, we excluded height from our index, although it can affect profitability. Straw yield is a function of height (Engel et al., 2003); thus, for growers who profit from straw, height would have a positive EV. Conversely, for growers who plant double‐crop soybeans and do not use or sell wheat straw, height would have a negative EV due to the impact of wheat residue on double‐crop soybean establishment.

Comprehensive EV tables, like what is available in dairy cattle breeding (VanRaden et al., 2025), could be developed for wheat breeding with additional research. As demonstrated for TW, an economic weight for DON could be derived using the discount schedules that grain buyers impose. However, it is important to bear in mind that DON phenotyping is typically conducted within an inoculated and misted nursery representing a severe FHB epidemic. Studies to quantify the relationship between genetic variation in DON concentrations observed in such nurseries and that expressed under production conditions may be needed to derive an appropriate economic weight. For lodging tolerance, an economic weight could be derived based on research examining all costs associated with different degrees of lodging across a representative set of environments. This may not be practically feasible given the sporadic nature of lodging and difficulty of quantifying some of its economic impacts. Traits for which EVs cannot be readily determined could be assigned a weight based on desired gains (Pešek & Baker, 1969) to provide an immediate solution.

Regardless of the type of weight used, defining a breeding objective requires a nuanced understanding of production practices and scenarios within the TPE. In wheat, production and pricing systems are region‐specific and can vary widely even within a region. This can result in system‐specific EVs for the same trait (Wolfová et al., 2005), potentially requiring multiple sets of economic weights to adequately represent a given TPE. Economic weights will also vary temporally due to the influence of genetic and nongenetic trends caused by genetic improvement, changes in the environment, and improvements in management. Knowledge of these trends could inform economic weights to ensure that genetic improvement is directed toward future production needs.

In this study, the selection index was developed for parent selection, where the objective is to maximize expected genetic gain in the next generation based on additive genetic merit. In contrast, when the objective is to advance lines as future commercial products, total genetic value based on additive + nonadditive genetic merit should be considered to evaluate product performance. The selection procedure would then involve a combination of index selection and independent culling to meet required thresholds (Werner et al., 2024). Given that our work aims to optimize parent selection for improving the breeding population, a next step would be to integrate complementary strategies such as optimum contribution selection (Woolliams et al., 2015), with MTME modeling to maximize genetic gain while maintaining genetic variation in the key traits over time. This would enhance and help secure the long‐term sustainability of genetic gain for the breeding program.

## 5 CONCLUSIONS

This study demonstrates that combining a genomic‐based SHI with an MTME model and empirically derived economic weights can improve selection efficiency in wheat. The optimal MTME model explained over 91% of the additive variance and achieved high prediction accuracy for all traits. The advantage of the MTME model over the STME model was clear based on outcomes of index selection. Compared to the STME, the MTME‐based index selection delivered higher predicted economic returns and achieved genetic gains in GY, TW, and maturity that were more consistent with the breeding objectives. Use of an MTME model also captured environment‐specific genetic correlations between traits, aiding our understanding of how local stress conditions impact trait relationships. Overall, this study serves as a useful case example for how a regional breeding program can optimize multi‐trait selection to accelerate genetic gain in total net merit.

## AUTHOR CONTRIBUTIONS

**Lucas Berger Munaro**: Data curation; formal analysis; investigation; methodology; software; visualization; writing—original draft; writing—review and editing. **Daniel J. Tolhurst**: Methodology; software; writing—review and editing. **Jessica E. Rutkoski**: Conceptualization; funding acquisition; methodology; project administration; resources; supervision; writing—review and editing.

## CONFLICT OF INTEREST STATEMENT

The authors declare no conflicts of interest.
